# Nanoscopy of the cellular response to hypoxia by means of fluorescence resonance energy transfer (FRET) and new *FRET *software

**DOI:** 10.1186/1757-5036-3-5

**Published:** 2010-03-05

**Authors:** Christoph Wotzlaw, Silke Gneuss, Rebecca Konietzny, Joachim Fandrey

**Affiliations:** 1 Institut für Physiologie, Universität Duisburg-Essen, D-45122 Essen, Germany

## Abstract

**Background:**

Cellular oxygen sensing is fundamental to all mammalian cells to adequately respond to a shortage of oxygen by increasing the expression of genes that will ensure energy homeostasis. The transcription factor Hypoxia-Inducible-Factor-1 (HIF-1) is the key regulator of the response because it coordinates the expression of hypoxia inducible genes. The abundance and activity of HIF-1 are controlled through posttranslational modification by hydroxylases, the cellular oxygen sensors, of which the activity is oxygen dependent.

**Methods:**

Fluorescence resonance energy transfer (FRET) was established to determine the assembly of the HIF-1 complex and to study the interaction of the α-subunit of HIF-1 with the O_2_-sensing hydroxylase. New software was developed to improve the quality and reliability of FRET measurements.

**Results:**

FRET revealed close proximity between the HIF-1 subunits in multiple cells. Data obtained by sensitized FRET in this study were fully compatible with previous work using acceptor bleaching FRET. Interaction between the O_2_-sensing hydroxylase PHD1 and HIF-1α was demonstrated and revealed exclusive localization of O_2_-sensing in the nucleus. The new software *FRET *significantly improved the quality and speed of FRET measurements.

**Conclusion:**

FRET measurements do not only allow following the assembly of the HIF-1 complex under hypoxic conditions but can also provide important information about the process of O_2_-sensing and its localisation within a cell.

**MCS codes:** 92C30, 92C05, 92C40

## 1. Background

Oxygen deprivation of the tissues endangers energy supply and thus function and survival of the cells. Hypoxia, defined as a state of reduced oxygen tension (PO_2_), develops when the demand for oxygen exceeds supply. To restore oxygen and thus energy homeostasis an adequate response requires the coordinated expression of genes that control tissue perfusion and oxygen capacity of the blood, glucose uptake and anaerobic glycolysis [1]. The key transcriptional regulator of the genomic response to hypoxia is the transcription factor complex Hypoxia-Inducible Factor-1 (HIF-1) which supposedly regulates about 5% of the human genome [2]. HIF-1 is formed as a dimer of oxygen-regulated α-subunits (HIF-1α or HIF-2α) and a constitutive nuclear subunit HIF-1β [3]. Under well oxygenated conditions (normoxia) three enzymes containing prolyl hydroxylase domains (PHD1, PHD2 and PHD3) posttranslationally modify HIF-α subunits. The hydroxylated α-subunits are recognized by the von Hippel-Lindau protein (pVHL) which recruits an E3 ubiquitin ligase to polyubiquitinate hydroxylated HIF-α subunits. Subsequently, ubiquitinated HIF-αs will be degraded by the 26S proteasomes [1].

Under hypoxia, HIF-1 can accumulate because PHDs require oxygen and their activity is greatly reduced under low PO_2_. Under hypoxic conditions stabilized HIF-αs translocate into the nucleus by not yet defined mechanisms. It is believed that they immediately dimerize with HIF-1β and recruit the transcriptional co-activators p300/CBP to the C-terminal activation domain (C-TAD) of HIF-αs [3]. This recruitment is also O_2_-sensitive because the factor inhibiting HIF-1 (FIH-1), an asparagyl hydroxylase, impedes binding of co-activators under normoxia due to O_2_-dependent hydroxylation of an asparagine residue in HIF-1αs [4]. Thus, HIF-1 is controlled both on the level of abundance and *trans*-activity of its α-subunits by O_2_-dependent hydroxylases that act as cellular oxygen sensors.

Imaging the components of the oxygen sensing mechanisms is difficult. We have previously localized the nuclear distribution of HIF-1α and of the O_2_-sensors PHD1, PHD2, PHD3 and FIH-1 [5,6]. So far, it has not been successful to crystallize full length HIF-αs and HIF-1β or even the HIF-1 complex to obtain information of its structure and potential changes within the complex upon transition from normoxia to hypoxia and vice versa. Only partial structures of PAS domains B (PAS is an acronym for **P**eriod-**A**ryl-Hydrocarbon receptor nuclear translocator-**S**ingle minded, the founders of this transcription factor family) which is present in HIF-αs and HIF-1β were unravelled [7]. It was proposed that within the HIF-1 complex the PAS B domain of HIF-α and HIF-1β are orientated in an anti-parallel way to form the HIF-1 dimer [7]. This is unusual with respect to other members of the PAS family [8].

Since optical resolution, however, is at best approximately 200 nm, light microscopy does not allow drawing conclusions with respect to the HIF-1 complex and protein-protein interaction of its subunits. To analyze the assembly of the HIF-1 complex we successfully applied fluorescence resonance energy transfer (FRET) [9]. To this aim HIF-1 subunits were labeled with enhanced cyano (ECFP) or yellow (EYFP) fluorescent protein [10]. We were able to fit our FRET data with the above mentioned model by Card et al. to support the notion that N- and C-termini of HIF-1α and HIF-1β make rather close contact [10].

Nevertheless, the oxygen sensing process itself remains enigmatic. It is still unresolved in which cellular compartments HIF-αs are degraded at higher oxygen concentrations. Very recent attempts to co-localize HIF-1α and PHDs by the use of fluorescent fusion proteins of HIF-subunits have led to the suggestion that cell-specific differences exist with respect to the localization of the interaction of HIF-α and the O_2_-sensors [11]. In addition to the uncertain subcellular localisation of the interaction, our understanding how the complex of the O_2_-sensors, i.e. the PHDs, and HIF-αs is formed, is fragmentary. In a very recent study it was suggested that PHD2 forms a homotrimer with a head-to-tail arrangement of single PHD2 molecules to constitute the active enzyme complex [12]. This model is based on crystal structure data which would well explain why PHD2 is isolated as part of a complex much larger than one PHD2 molecule alone. It still remains to be demonstrated if and where the trimeric complex is formed in living cells.

Thus, although HIF is recognized as the master regulator of O_2_-homeostasis, we still lack detailed knowledge on several key aspects of regulation which is critically needed in view of the importance of HIF-1 for tumor biology and the pathophysiology of ischemic disease.

To study the assembly of the HIF-1 complex under hypoxic conditions and the interaction between PHDs and HIF-1α the microscopic FRET technique was used [13,14]. In the literature a couple of methods for detection of FRET between fluorophores attached to interacting protein partners are described [15]. Among them are the technically challenging fluorescence lifetime analysis of the fluorophores [16], acceptor bleaching and sensitized FRET techniques [17]. The latter provides the opportunity of protein-protein interaction analysis without destruction of the acceptor.

Herein, new software is presented that is easy to use for physiologists and biologists studying intracellular protein-protein-interaction in their specific field of research. To this aim we want to provide software that should be able to handle most inherent problems like heterogeneous illumination of the scanned field by the laser when FRET is performed on the microscope and false positive FRET signals. During scans in each experiment non-specific energy transfer originating from unspecific dimerization of fluorophores or random collision should be measured because non-specific energy transfer determines the biological and also technical limits of FRET detection in a given system [18,19]. Areas of overexposure due to precipitation or clumping of fluorophores and areas where no co-localization is found or where fluorescent signals are too weak for reliable FRET should be excluded from the evaluation. Actual bleed-through values which may be due to unspecific activation of one fluorophore by the laser specific for the other partner fluorophore should be automatically and regularly determined. Finally, the software should display the results of FRET as the sigmoid relation between the ratio of acceptor to donor fluorescence and FRET efficiency over the whole range of donor/acceptor ratios. We finally used our new system to determine the proximity of the two HIF-1 subunits and to prove PHD-HIF-1α interaction in the cell nucleus.

## 2. Methods

### 2.1 Cell culture

The human osteosarcoma cells (U-2OS) were grown in Dulbecco's Modified Eagle's Medium (DMEM with 4.5 g/l glucose, L-glutamine, and pyruvate; all from Invitrogen GmbH, Karlsruhe, Germany) supplemented with penicillin (100 U/ml), streptomycin (100 μg/ml; Invitrogen GmbH, Karlsruhe, Germany) and 10% fetal bovine serum (FBS; Biocompare, South San Francisco, USA) in cell culture flask (75 cm^2 ^bottom area; Greiner Bio-One GmbH, Solingen, Germany) in a normoxic atmosphere of 21% O_2_, 74% N_2 _and 5% CO_2 _(by vol.) in a Heraeus incubator (Heraeus, Hanau, Germany). For human embryonal kidney cells (HEK293), DMEM/F-12+GlutaMAX™-I supplemented with 10% fetal calf serum, 100 U/ml penicillin and 100 μg/ml streptomycin was used. For FRET experiments cells were grown on glass-bottom cell culture dishes, 3.5 cm in diameter (WillcoWells BV, Amsterdam, Netherlands) with central glass inserts that allow all standard formats of microscopy including UV light excitation.

### 2.2 Plasmids and transfection

For transient transfection plasmid DNA was mixed with Fugene6 (Roche, Mannheim, Germany) and suspended in appropriate volumes of DMEM for each culture dish. Cells were transfected with the vectors for 24 h, medium was renewed for another 24 h and then again 1 h before starting the experiments. The following plasmids were used which have been described in detail before [10]: HIF-1α fused to enhanced cyan fluorescent protein (ECFP; Clontech GmbH, Germany) yielding ECFP-HIF-1α-ECFP- HIF-1α and HIF-1β fused to enhanced yellow fluorescent protein (EYFP; Clontech GmbH, Germany) yielding EYFP-HIF-1β, respectively. Prolyl hydroxylase 1 (PHD1) was fused to EYFP resulting in PHD1-EYFP.

Hypoxic incubations were done in an atmosphere of 1% O_2_/5% CO_2_/94% N_2 _at 37°C in a chamber specially designed for observation of living cells on microscope stage under the conditions of controlled temperature, humidity and gas composition, e.g. O_2 _and CO_2 _(Luigs & Neumann, Ratingen, Germany) [10]. Gas concentrations were individually adjusted by a gas mixing device (Newport Spectra-Physics GmbH, Darmstadt, Germany) connected to the chamber.

For sensitized FRET analysis of protein-protein-interactions a standard inverted confocal microscope with objective lenses plan Apochromat 40× (Nikon GmbH, Düsseldorf, Germany) was used with one excitation laser line of 444 nm for ECFP (termed 'donor' for FRET measurements) and a second laser between wavelength of 532 nm for EYFP (termed 'acceptor' for FRET) excitation. For FRET two band pass emission filters 480/40 and 565/40 nm (AHF AG, Tübingen, Germany) were used.

### 2.3 Correction for shading

Automatic shading correction was implemented using the plugin by Wayne Rasband (NIH) for ImageJ using the formula for calculating the corrected image B': B' = B*(M/Y) where B is the image which has to be corrected, M is the mean of the shading image, Y is the shading image.

### 2.4 Minimizing errors due to chromatic aberration

In addition, during the scan procedure chromatic aberration was minimized. To this aim the objective lens was moved 0.5 μm by a piezo stepper during exciting with the EYFP laser (532 nm) in relation to the excitation with the ECFP laser (444 nm).

### 2.5 Calibration

To determine the bleed-through and calibrate the systems ECFP fluorescence which is normally detected in the range of 460-500 nm after excitation with the ECFP laser (444 nm) was determined in the emission channel for the acceptor EYFP from 545-585 nm. This was done with cells that only contain the ECFP construct and is called donor bleed-through (donor BT) when related to the detection of ECFP signal in the specific EYFP emission channel. Subsequently, the EYFP fluorescence was recorded after excitation with the ECFP laser in cells that were only transfected with EYFP. This is called acceptor bleed-through (acceptor BT) when related to the fluorescence signal in the specific EYFP channel after excitation with EYFP laser. Both measurements are required since during the experiments cells will be co-transfected with both fusion proteins and both lasers will be used. We are using a standard correction method, originally described in [13].

### 2.6 Data acquisition

**Figure 1:**
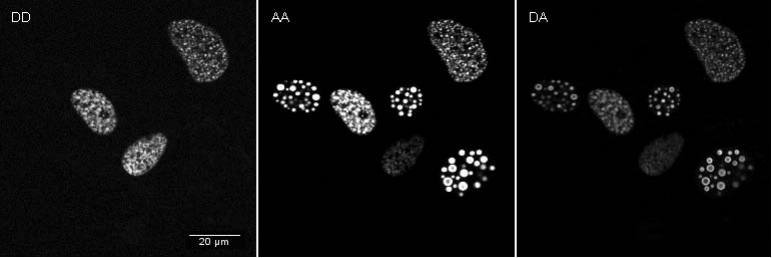
**Data acquisition of FRET**. Osteosarcoma cells U-2OS were transfected with plasmids expressing the donor molecule ECFP-HIF-1α and the acceptor EYFP-HIF-1β. 48 h later cells were scanned for fluorescence and FRET was measured. Data for the panels in the upper row were recorded for panel **DD **by donor excitation- donor detection, for panel **DA **by donor excitation- acceptor detection and in panel **AA **by acceptor excitation-acceptor detection.

To determine protein-protein interaction by FRET cells were transfected with ECFP-HIF-1α (donor) and EYFP-HIF-1β (acceptor). First, the cells were excited with the donor laser (444 nm) and the emission was detected in the donor channel (see panel DD for donor excitation-donor detection in Figure 1). Then emission in the acceptor channel after donor laser excitation was determined (see panel DA for donor excitation-acceptor detection in Figure 1). Finally, the cells were excited with the EYFP laser (532 nm) and emission is detected in the acceptor channel (panel AA for acceptor excitation-acceptor detection in Figure 1). First we calculated Net FRET (nF) according to [13]:

nF = DA - DD* BT_Donor _- AA*BT_Acceptor_, where DA (donor excitation - acceptor fluorescence), DD (donor excitation - donor fluorescence), and AA (acceptor excitation - acceptor fluorescence) are intensities in each region of interest (ROI) with FRET, EYFP, and ECFP filter sets, respectively. BT_Donor _is the percentage of CFP bleed-through, and BT_Acceptor _the percentage of EYFP bleed-through under the FRET filter set. There were no signals from ECFP with EYFP filter sets and vice versa.

**Figure 2:**
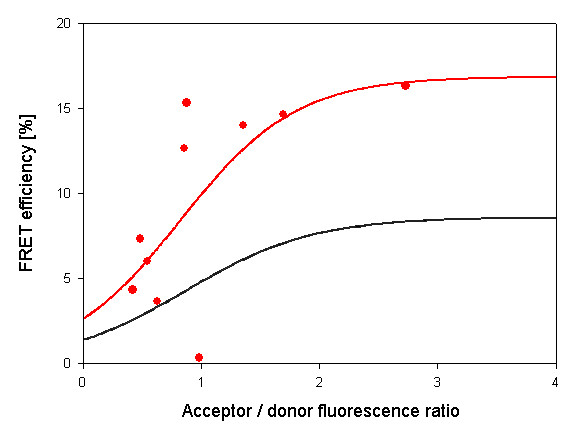
**FRET evaluation of ECF-HIF-1α and EYFP-HIF-1β interaction**. Osteosarcoma cells U-2OS were transfected with plasmids expressing the donor molecule ECFP-HIF-1α and the acceptor EYFP-HIF-1β. 48 h later cells were scanned for fluorescence and FRET was determined. The black curve indicates random FRET values as described in detail in Figure 3. Red filled circles show FRET efficiency values from individual cells which result in mean FRET efficiency for HIF-1α/HIF-1β as indicated by the red curve.

Assuming that the value of non-radiation relaxation processes during/after FRET is negligible we calculated FRET efficiency for each pixel in the images by the following formula:

According to Vamosi et al. [20] the factor α relates to the ratio of fluorescence quantum yields and the detection efficiencies of both dyes (in our case ECFP and EYFP). In our analysis we simplified the evaluation by setting α to 1 because results of sensitized FRET experiments between fluorophore labelled HIF-1α and HIF-1β subunits (Figure 2) show comparable plateau values as in acceptor bleaching experiments published earlier [10]. For evaluation, only panels from registration DD and AA with pixel values that are above and below a respective threshold value were used. As an orientation for determination of these threshold values we analyzed several images which were taken for determination of BT_Donor _and BT_Acceptor _values. The factor α was included according to Vamosi et al. [20] which relates to the ratio of fluorescence quantum yields of two different fluophores (in our case ECFP and EYFP). In our analysis we simplified the evaluation by setting α to 1. Over a wide range of fluorescence signal the BT values are constant. Below signal strength of about 10% the photomultiplier dynamic range the BTx values vary considerably. Above signal strength of 90% of the photomultiplier dynamic range the BT values show a tendency to exponentially increase with fluorescence intensity of the fluorophore. Non-transfected cells show very weak auto-fluorescence signals in both the donor and the FRET channel which are much lower than any fluorescence intensities from fusion proteins. These low intensities were measured in separate but not considered in image analysis. Only intensities above these values were included in the analysis.

For background determination the software will put thirty 15 × 15 pixel squares at random position over the image. The square with the lowest mean grey value will be defined as background. The background correction has been implemented into ImageJ. Overexposed areas, i.e. areas of very high fluorescence signal strength, were not evaluated because of the process of exclusion of fluorescence values from the evaluation process described above. Due to a sigmoid relationship between the FRET efficiency and the ratio of acceptor to donor molecules we did not normalized nF. Instead we show the graphical evaluation the FRET efficiency data.

The program was written as a macro to be implemented into the program *Image J *which is freely available from the National Institutes of Health, USA http://rsbweb.nih.gov/ij.

## 3. Results

### 3.1 Determination of non-specific energy transfer

**Figure 3:**
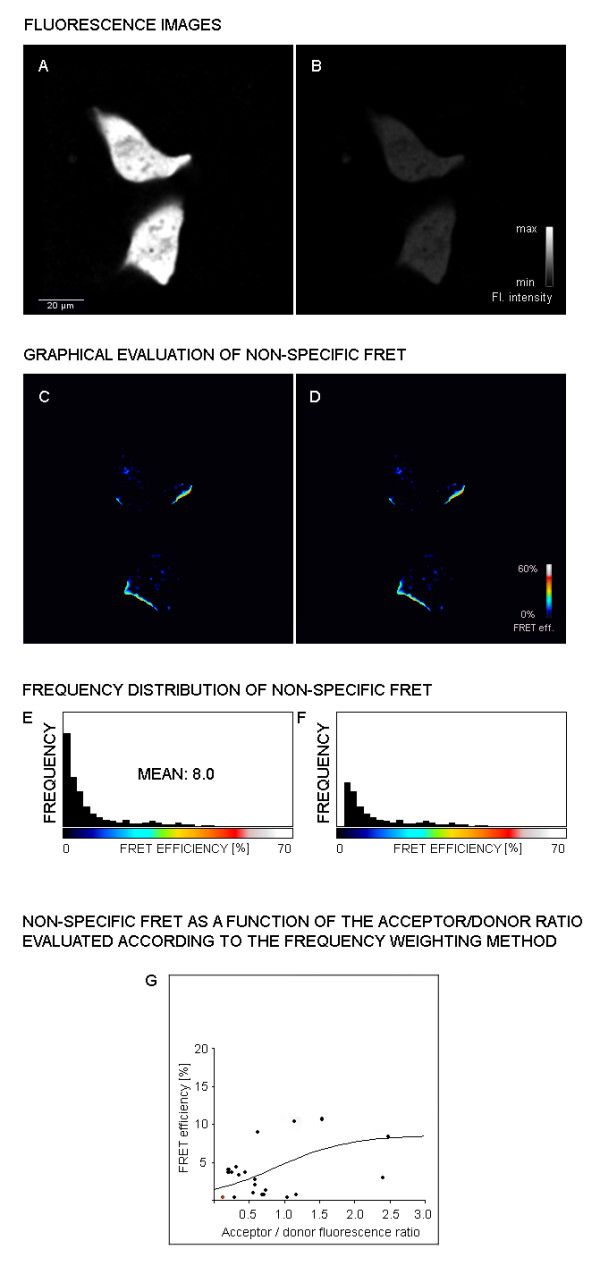
**Determination of random FRET**. Osteosarcoma cells U-2OS were transfected with plasmids expressing the fluorophores ECFP- and EYFP only. 48 h later cells were scanned for fluorescence and random FRET was determined. Panel **A: **ECFP fluorescence after donor excitation; panel **B**: EYFP acceptor fluorescence after acceptor excitation; panel **C: **non-specific energy transfer fluorescence encoded in pseudo colors and panel **E**: corresponding frequency distribution of FRET efficiencies; panel **D: **non-specific energy transfer fluorescence encoded in pseudo colors after cutting of efficiency values below a certain threshold and panel **F**: corresponding frequency distribution of FRET efficiencies; panel **H: **resulting non-specific, random FRET curve after weighing FRET efficiencies for their relative frequency.

**Figure 4:**
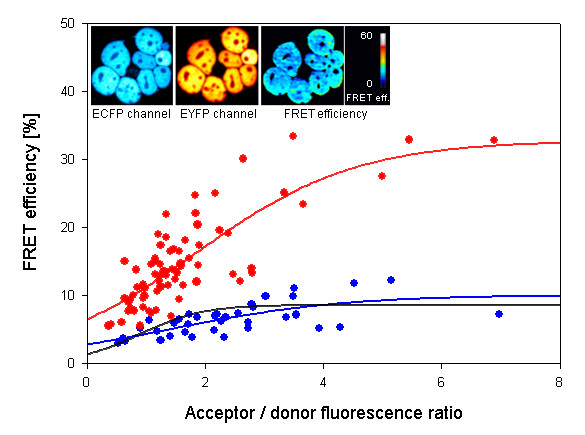
**FRET evaluation of ECF-HIF-1α and PHD1-EYFP interaction**. Embryonic kidney cancer cells HEK 293 were transfected with plasmids expressing the donor molecule ECFP-HIF-1α and the acceptor PHD1-EYFP. 48 h later cells were scanned for fluorescence and FRET was determined. The black curve indicates random FRET efficiency values as described in detail in Figure 3. Filled circles show FRET values from individual cells which result in mean FRET efficiency for HIF-1α/PHD1 as indicated by the red and blue curves. Values for the red curve were obtained after treatment of the cells with the PHD1 inhibitor dimethyloxalylglycine (0.5 mM for 24 h) while the blue curve shows values from untreated controls. The inserts show donor (ECFP-HIF-1α) and acceptor (PHD1-EYFP) fluorescence and the resulting FRET efficiency exclusively located in the nuclei of the cells.

To obtain reliable FRET results non-specific energy transfer values have to be taken into consideration. Cells were only transfected with the vectors for ECFP and EYFP but not with vectors for fusion proteins with HIF-1 subunits. Cells were transfected with vectors containing either ECFP or EYFP alone and fluorescence of ECFP (Figure 3, panel A) and EYFP (Figure 3, panel B) was recorded. Panel C shows non-specific energy transfer which is particularly prominent at the edge of the cells while low intensity non-specific energy transfer is found over the whole cell area. One way of handling with the fact of non-specific energy transfer is to set a FRET efficiency threshold. Values below this threshold value are then excluded from the visualization of FRET efficiency distribution (Panel D). Panel D, however, shows an almost identical picture as panel C. In panel E and panel F histograms of the frequency distribution of the different non-specific FRET is shown for the pictures from panel C and D respectively. Only a few very high values cause a mean efficiency of 8% in panel E; interestingly, cutting off values below a certain threshold (panel F) does not improve the situation because few high random FRET values shift the mean value to 11.8%. The program now avoids this problem by not simply cutting-off FRET efficiency values below a threshold, but weighing their values by frequency for each acceptor/donor ratio. By these means, less frequent outliers representing unusual high or low values will not be included in the evaluation. The result is shown in panel H where single FRET efficiencies are shown and the sigmoid curve resulting from the mean efficiencies at different acceptor/donor ratios. The values of this method for determining non-specific energy transfer were comparable to FRET data where one dimerizing FRET partner was unable to specifically interact due to a deletion of the interacting domain [21]. Determination of non-specific energy transfer efficiency was incorporated into the software and taken into consideration for all FRET measurements (also see Figures 2 and 4).

### 3.2 Evaluation of ECFP-HIF-1α and EYFP-HIF-1β FRET

**Figure 5:**
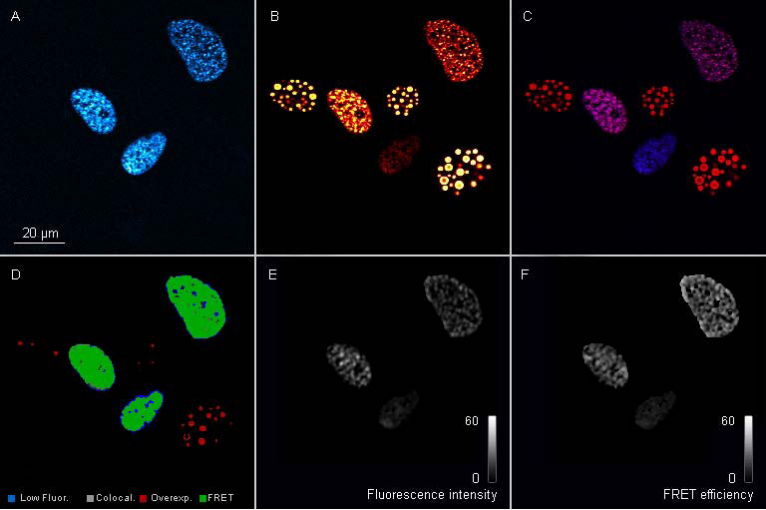
**Typical recording of a FRET measurement**. Osteosarcoma cells U-2OS were transfected with plasmids expressing the donor molecule ECFP-HIF-1α and the acceptor EYFP-HIF-1β. 48 h later cells were scanned for fluorescence and FRET was determined. Panel **A: **ECFP-HIF-1α donor fluorescence after donor excitation; panel **B**: EYFP-HIF-1β acceptor fluorescence after acceptor excitation; panel **C**: overlay of images in panels A and B; panel **D**: areas of co-localization (from panel C), very low signal (in blue) or overexposed areas (in red) and areas with FRET (from panel E); panel **E: **EYFP-HIF-1β acceptor fluorescence after donor excitation = FRET fluorescence intensity encoded in grey values as indicated by the bar scale; panel **F**: FRET efficiency encoded in grey values as indicated by the bar scale.

The results of the FRET measurements in a single nucleus of an U2OS cell transfected with ECFP-HIF-1α and EYFP-HIF-1β fusion proteins are shown in the following panels in Figure 5. Panel A shows the donor emission, in this case, from the ECFP-HIF-1α fusion protein. Panel B shows the acceptor fluorescence, here EYFP-HIF-1β. The overlay of both channels results in panel C. From this, in panel D co-localization areas were calculated which indicate the areas where FRET should be possible. In this panel, areas with very low signals in the donor or acceptor channel (fluorescence <10% of the dynamic range of used fluorescence detectors) are shown in blue whereas overexposed areas were encoded in red (fluorescence >90% of the dynamic range of used fluorescence detectors). Both blue and red areas are not included in the evaluation. This process increases the reliability of FRET determination. Panel E then shows FRET fluorescence as the result of the radiation less energy transfer from ECFP-HIF-1α to EYFP-HIF-1β . Panel F contains the calculated FRET efficiency.

Our new software was then used to determine the proximity of the α- and β-subunits of HIF-1 as shown in Figure 2. FRET was readily detectable in cotransfected U2OS osteosarcoma cells and allowed to calculate the distance between the two fusion proteins with about 6.4 nm assuming that the radiation less energy transfer only takes place between two molecules and without any competition between molecules of the same form [22]. This result is in good agreement with our previous study using FRET with the acceptor bleaching method [10].

### 3.3 Interaction of HIF-1α with PHD1

In addition to understanding the assembly of the HIF-1 complex by FRET, it is important to determine the interaction between the oxygen sensors PHDs and HIF-1α subunit. Posttranslational hydroxylation of HIF-1α by PHDs is the key step in oxygen dependent regulation of the whole HIF-1 complex. Since previous experiments had shown different compartmentalization of oxygen sensors, i.e. PHD1 localized to the nucleus and PHD2 and 3 mainly localized to the cytoplasm [5], the localized FRET is also of considerable interest to understand oxygen sensing. *FRET *software and FRET were applied to determine this interaction. To this aim, HEK293 cells were transfected with ECFP-HIF-1α as the donor molecule and PHD1-EYFP as the acceptor molecule. Cells were co-transfected and FRET efficiency was determined using *FRET *software. The results are shown in Figure 4. Random FRET is shown as a black line in this Figure. Transfection with the two fusion proteins revealed only moderate FRET efficiency, as indicated by the blue symbols and the blue sigmoid curve in Figure 4. However, when dimethyloxalylglycine (DMOG), an inhibitor and pseudo-substrate of PHD1, was added which causes inhibition of the enzyme and accumulation of HIF-1α, much higher FRET efficiencies were obtained as shown by the red symbols and the red sigmoid curve in Figure 4. FRET was exclusively found over the nucleus as shown by the inserts in Figure 4, clearly demonstrating the advantage of this new software to reliably determine FRET efficiency with spatial resolution. Furthermore, these data indicate that oxygen sensing by PHD1 is localized within the nuclear compartment due to the nuclear interaction of PHD1 with HIF-1α .

## 4. Discussion

To maintain oxygen homeostasis within cells and the tissue is fundamental for the human body. Common to the pathophysiology of all ischemic diseases is a lack of oxygen in the cells that causes impaired function or even cell death. Many of these examples include necrosis of cardiomyocytes after myocardial infarction or necrosis and apoptosis of neural cells after stroke [23]. In addition, hypoxia plays a fundamental role in tumor biology. It has been known for many years that hypoxic regions in tumors develop because tumor cells grow much faster than the oxygen supplying capillaries [24]. In fact, capillary growth due to an increased oxygen demand has been the hallmark of many cancers and used for diagnosis, e.g. in malignant glioblastomas. In recent years, it has been recognized that not only an increase in capillary growth, angiogenesis, but other tumor-specific events depend on hypoxia [25]. Apoptosis resistance, metastases and also genetic instability have now been all attributed to activation of hypoxia-inducible gene expression under control of hypoxia-inducible factor-1 (HIF-1) [25,26]. While HIF-1 also is in control of angiogenesis and many genes that stimulate and control angiogenesis, activation of HIF may also be caused by the tumor phenotype. This can then lead to induction of glycolytic enzymes despite sufficient oxygen concentration. This phenomenon known as the Warburg effect is another hallmark of malignant cells and an excellent example how malignant cells make use of physiological adaptation mechanisms to gain a growth advantage over adjacent host tissue [27].

Not only since HIF-1 has been named "the master regulator of oxygen homeostasis" [28] attempts have been made to therapeutically intervene with HIF-1 activation. This may in cases of ischemic diseases be an increased activation of HIF and thus, according to the control mechanisms, a stabilization of HIF-α subunits [29]. On the other hand, inhibition of the HIF complex may be required in malignant diseases to decrease the HIF-1-dependent response of tumor cells. For such fundamental processes at oxygen sensing and activation of HIF-1, initial studies have to be performed in cell culture systems in vitro. These systems would provide a well-controlled environment to study changes in oxygenation with respect to activation of the HIF-1 complex. On the other hand, even *in vitro *systems may show heterogeneity of oxygen distribution which complicates experimental setups [30].

So far, molecular biology methods have been applied to study activation of the HIF-1 complex. We have recently introduced fluorescence resonance energy transfer (FRET) to study the assembly of the HIF complex [10]. Like in the present study, subunits of HIF-1 were labeled with fluorophores and then transfected into living cells to study the activation and assembly of the HIF complex. This system relied on the microscopy of living cells in a well-controlled observation chamber that allowed specification of oxygen tension, temperature and CO_2 _for buffering [10]. This system proved to be very useful since physiological regulation of HIF-1, i. e. accumulation of the α-subunit under hypoxic conditions, was mostly conserved. Both HIF-1α- and -β-subunits bind to DNA through their basic helix-loop-helix-region in the N-terminal end of the protein [31]. These parts have also been proposed for dimerisation of the subunit. FRET analysis revealed a very close assembly when fusion proteins bound to DNA [10]. Recent evidence using deletion constructs in this DNA binding domain revealed that assembly and close vicinity of the subunits with a distance of only 6.4 nm depends on DNA binding (R. Konietzny and J. Fandrey, unpublished). On the other hand, the C-terminal end of HIF-1α is important for binding additional co-activators and scaffolding proteins that are required for the fully active HIF-1 complex [1,31]. When both C-terminal ends were labeled with fluorophores, the distance was still close (6.7 nm), but one may hypothesize that additional factors bound to this end of the protein might push the two subunits further apart than at the N-terminus where DNA binding affects proximity of the proteins. Interestingly, an attempt to disrupt FRET by labeling opposite ends of the protein revealed that FRET was still observed between the end-terminal DNA bound part of HIF-1 and its the C-terminal part, because FRET calculations revealed a distance of 7.4 nm [10]. These results were unexpected but obtained at a time when initial attempts were made to reveal the protein structure by X-ray diffraction analysis [7]. The HIF subunits hardly go into solution and crystallization of a single subunit or the whole complex has not been achieved yet. Parts of the HIF protein, the PAS-A and PAS-B domains, where PAS is an acronym delineating the family of highly related transcription factors Period, ARNT and Single-minded PAS domains [32]. The group of Gardner et al. was able to crystallize parts of these HIF proteins, in particular PAS-B structure, and found that upon assembly of the HIF complex PAS-B domains will form an antiparallel orientation [7]. Taking this information and combining it with our in vivo obtained FRET data, makes it likely that the HIF complex is rather compactly formed in its DNA-bound form which brings the C-terminus of the HIF complex close to DNA where it could interact through co-activators with other DNA-binding proteins, for example hepatic nuclear factor-4 (HNF-4), which is required for tissue-specific expression of the HIF target gene erythropoietin [33]. All in all, these FRET measurements provided for the first time the required in vivo data of the HIF complex and were also fully compatible with the only partly revealed X-ray structure of the PAS domains. One inherent problem of these in vitro studies, however, is the above-mentioned heterogeneity in oxygen concentration that may also occur in cell culture. We have therefore set up the present system where multiple cells can be scanned at the same time to account for these differences in the experimental setup. Moreover, based on our initial findings, the specificity of FRET of the HIF complex was confined and therefore allowed the use of sensitized FRET. Herein, we show that several cells can be semi-automatically scanned for fluorescence of the respective FRET partners which then allows areas of co-localization. The direct determination of sensitized FRET then provides the examiner with information about the degree of FRET in the different areas in cells over a whole range of many cells in one individual experimental setup. This allows for the first time to get an impression about the heterogeneity of HIF activation in cells in culture and also in cells growing in close clusters. In addition, the system can be used for dynamic studies since we do no longer rely on acceptor bleaching which would inevitably destroy one FRET partner. Our system is also suitable to study attempts to affect the HIF-1 complex assembly which appears to become an attractive target to modify activation of the HIF-1 complex [34].

The precise localization of cellular oxygen sensing has been enigmatic. Mostly based on experiments with cellular extracts, but also immunohistochemical staining studies have revealed that the cellular oxygen sensors PHD2 and PHD-3 were predominantly localized to the cytoplasm. In contrast, PHD1 was exclusively detected in the nucleus [5]. Recent studies in which cells had been modulated in their oxygen sensing capacity by nitric oxide treatment have shown that the localization of PHD2 can be shifted from the cytoplasm to the nucleus [35]. Cell fractionation experiments unequivocally showed that activity of prolyl hydroxylases is found in the nuclear compartment [35,36].

Our data herein for the first time show that interaction between the oxygen sensor PHD1 and HIF-1α protein takes place in the nucleus. Interestingly, the interaction was only detected when the enzymatic activity of PHD1 was inhibited by the pseudo substrate inhibitor DMOG (Figure 4). It has been hypothesized that inhibition of the enzyme may increase the contact time between PHD and the substrate [37]. In our case, this would indicate that the interaction between PHD1 and HIF-1α is too short-lived to be detected by FRET without inhibition but last long enough when the enzyme is inhibited. This hypothesis clearly needs further experimental evidence. With respect to the localization of FRET, so far mitochondria or the endoplasmatic reticulum have been considered as a place for cellular oxygen sensor activity. Two very recent studies indicate that adapter proteins may interact with PHD2 oxygen sensing enzyme to either determine the stability of PHD2 protein [21] or to affect activity [38]. Interestingly the study by Barth et al. provided evidence obtained by FRET measurements that an endoplasmatic reticulum bound protein interacts with PHD2 sensors only if correctly localized and orientated within the cell [21].

It has not been fully resolved yet, how this compartmentalization contributes to oxygen sensing but recent data indicate that nuclear specific activity of prolyl hydroxylases may be higher than cytoplasmic in many cell types [36]. Our data herein provide a new tool to study the localized interaction of oxygen sensors and the target protein HIF-1α in the nucleus. With respect to potential targeting of HIF or the oxygen sensing process itself for therapeutical intervention the nuclear localization has implication with respect to a strategy: It will be more difficult to target nuclear structures by small compounds. On the other hand, mechanisms of exclusion or transport into the nucleus are required for nuclear localization of the oxygen sensors and may thus be a valuable target.

## 5. Conclusion

We have set up new FRET software that allows the detection of several cells at the same time and evaluate FRET efficiency as well as localization of FRET. With respect to oxygen sensing this will help to clarify the assembly process of the HIF-1 complex itself but also the mechanisms of compartment-specific cellular oxygen sensing with particularly focus on nuclear oxygen sensors.

## Competing interests

The authors declare that they have no competing interests.

## Authors' contributions

CW programmed the software *FRET*, performed experiments and contributed data; SG and RK performed experiments and contributed data; CW, SG and JF wrote the manuscript.

All authors have read and approved the final manuscript.
